# The role of autophagy in Parkinson’s disease: a gender difference overview

**DOI:** 10.3389/fphar.2024.1408152

**Published:** 2024-06-12

**Authors:** Laura Cucinotta, Deborah Mannino, Alessia Filippone, Adele Romano, Emanuela Esposito, Irene Paterniti

**Affiliations:** ^1^ Department of Chemical, Biological, Pharmaceutical and Environmental Sciences, University of Messina, Messina, Italy; ^2^ Department of Physiology and Pharmacology “V. Erspamer”, Sapienza University of Rome, Rome, Italy

**Keywords:** Parkinson’s disease, gender differences, autophagy, ATG (autophagy-related) proteins, leucin rich repeat kinase 2 (LRRK2)

## Abstract

Recent studies have demonstrated dysregulation of the autophagy pathway in patients with Parkinson’s disease (PD) and in animal models of PD, highlighting its emerging role in disease. In particular, several studies indicate that autophagy, which is an essential degradative process for the damaged protein homeostasis and the management of cell balance, can manifest significant variations according to gender. While some evidence suggests increased autophagic activation in men with PD, women may have distinct regulatory patterns. In this review, we examined the existing literature on gender differences in PD-associated autophagic processes, focusing on the autophagy related proteins (ATGs) and leucine rich repeat kinase 2 (LRRK2) genes. Also, this review would suggest that an in-depth understanding of these gender differences in autophagic processes could open new perspectives for personalized therapeutic strategies, promoting more effective and targeted management of PD.

## 1 Introduction

PD is one of the most common neurodegenerative diseases, characterized by an accumulation of α-synuclein (α-Syn) protein within dopaminergic neurons, carrying to neuronal death (Sveinbjornsdottir, 2016). Autophagic processes, responsible for removing damaged proteins and managing cell balance, play a key role in understanding the pathogenesis of the disease (Nechushtai et al., 2023). Recently, more studies have focused on gender disparities in the manifestation and progression of PD. In this context, gender variations in autophagic processes could contribute significantly to the different incidence and modes of response to the disease between men and women (Lista et al., 2011). This review aims to explore and synthesize the current evidence outlining gender differences in PD-associated autophagic processes, providing a clear and detailed overview of autophagic processes in relation to PD, with a specific focus on their variation by gender. By suggesting an in-depth study, it is possible to open up new perspectives in the personalization of therapies and differentiated management of PD, finally representing a significant contribution to research and clinical practice.

## 2 Gender differences in PD symptoms and progression

From the analysis of several studies on PD, there is clearly an interesting variety in the manifestations of the disease between men and women. In particular, it appears that the course and severity of symptoms differ, with some aspects being more prevalent in men than in women (Haaxma et al., 2007). In fact, research indicates that men have a significantly higher risk of developing PD, with a ratio ranging from 1.4 to 3.7 compared to women, however, women have a higher mortality rate (Dahodwala et al., 2016). On the other hand, it remains unclear how much this difference in risk affects disease progression. Abraham et al. (2019) suggest that sex influences the onset of the disease but appears to have minimal impact on its progression.

The reasons behind the higher incidence in men are not well known; several studies have therefore, focused on understanding the factors that might contribute to these gender differences (Augustine et al., 2015). They could depend on varying degrees of exposure to environmental risk factors, the influence of sex-specific hormones, or potential genetic factors (Lubomski et al., 2014). The research reported by Blauwendraat and collaborators stresses the importance of considering differential exposures to environmental risk factors, such as pesticides and heavy metals, that could contribute to the observed disparities between men and women (Blauwendraat et al., 2021).

Hormonal factors emerge as a key component in gender differences related to PD (Gillies et al., 2004; Brann et al., 2007). The presence of sex hormones, particularly estrogen, appears to play a significant role in modulating risk and disease progression. Studies such as those conducted by Coronel indicate that estrogens may exert neuroprotective effects on the nigrostriatal dopaminergic system through the inhibition of reactive glia and inflammatory cytokine cascade and also, may modulate monoamine oxidase (MAO), thereby influencing disease manifestation (Jurado-Coronel et al., 2018). The neuroprotective effect exerted by estrogen could be due to its ability to reduce inflammation, to promote the survival of dopaminergic nerve cells involved in PD, and ultimately neuroplasticity, helping to maintain brain function by offsetting the damage caused by PD. The role of testosterone in neurodegeneration may depend on several factors such as age and hormone concentration (Yan et al., 2021). However, more studies are needed to understand the exact correlation between hormones and neurodegeneration (Young and Pfaff, 2014).

In PD, the brain regions involved, such as the substantia nigra, may be exposed experience to a loss of nerve cells and, as a result, a reduction in Gray matter volume. This reduction was observed in regions closely related to motor control, but also in other brain regions involved in cognitive functions. Gray matter, made up of neurons that acts trough synapses, is involved in cognitive, sensory and motor functions, while white matter is composed of myelinated nerve fibers that play a key role in neuronal communication. Neuroanatomical differences between men and women, highlighted by Cai et al. (2023), further contribute to the complexity of the picture. Females have a greater relative Gray matter volume, while males have a greater relative white matter volume. Brain structure, including the size of the basal nuclei, appears to vary differentially between the sexes, introducing additional variables that could modulate the manifestation of PD (Cai et al., 2023). Analysing the ratio of Gray and white matter through neuroimaging techniques can provide valuable insights into the location and extent of brain degeneration at different stages of the disease.

Clinical heterogeneity between the sexes emerges clearly in symptoms and response to treatments (Georgiev et al., 2017; Bianco et al., 2023). Modulations in neurotransmitter distribution and receptor sensitivity may contribute to differences in PD motor symptoms between men and women. For example, dopamine is a neurotransmitter involved in the regulation of movement, and its deficiency is related to the motor symptoms of PD; changes in the function of dopaminergic receptors affect the transmission of nerve signals and the consequent manifestation of motor symptoms (Ramesh and Arachchige, 2023). Many studies have reported that in women, motor symptoms tend to emerge later and are characterized by reduced rigidity, tremor, and a greater propensity to develop postural instability (Cerri et al., 2019). In fact, women develop a dominant phenotype of more benign PD tremor associated with less severe motor impairment, whereas men develop a dominant phenotype of postural instability with gait disturbances (Reekes et al., 2020). Gender differences are not limited to motor symptoms; Coronel in his study points out that women are more susceptible to non-motor symptoms such as depression, constipation, loss of taste or smell, pain, and excessive sweating, while symptoms such as sexual dysfunction are more common in men (Jurado-Coronel et al., 2018).

Several studies reported sex differences in the efficacy, tolerability, and pharmacokinetics of PD treatments (Kompoliti et al., 2002; Martinelli et al., 2003; Shulman, 2007; Kumagai et al., 2014) and have been reported as having a greater risk of complications (Umeh et al., 2014).

The most common animal models of PD are often performed on male rodents (about 80%) and pathological findings between male and female animals are rarely compared. Bourque et al. (2023) show a gender difference in nigrostriatal degeneration in mouse models of PD, indicating greater MPTP-induced toxicity in males than females. Similar data have been confirmed by several other studies that have shown greater lesions in males than in females (Dluzen, 2000; Yu and Liao, 2000; Liu and Dluzen, 2007; Gillies and McArthur, 2010); however, in the case of lesions greater than 70%–80%, sex differences were abolished (Gillies et al., 2004; Gillies and McArthur, 2010) (Table 1).

**TABLE 1:** Main gender differences in PD.

Features	More prevalent in	Causes	Reference
*Incidence*	Men	Increased exposure to environmental risk factors, influence of sex-specific hormones, or potential genetic factors	Haaxma et al. (2007)
*Mortality rate*	Women	Differences in sex hormone exposure and sex chromosome effects	Dahodwala et al. (2016)
*Motor symptoms*	Men	Differences in the function of dopaminergic receptors which affect manifestation of motor symptoms	Cerri et al. (2019)
*Non-motor symptoms*	Women	Women are generally more susceptible to depression, fatigue and anxiety	Reekes et al. (2020)

## 3 The emerging role of autophagy in PD

**FIGURE 1:**
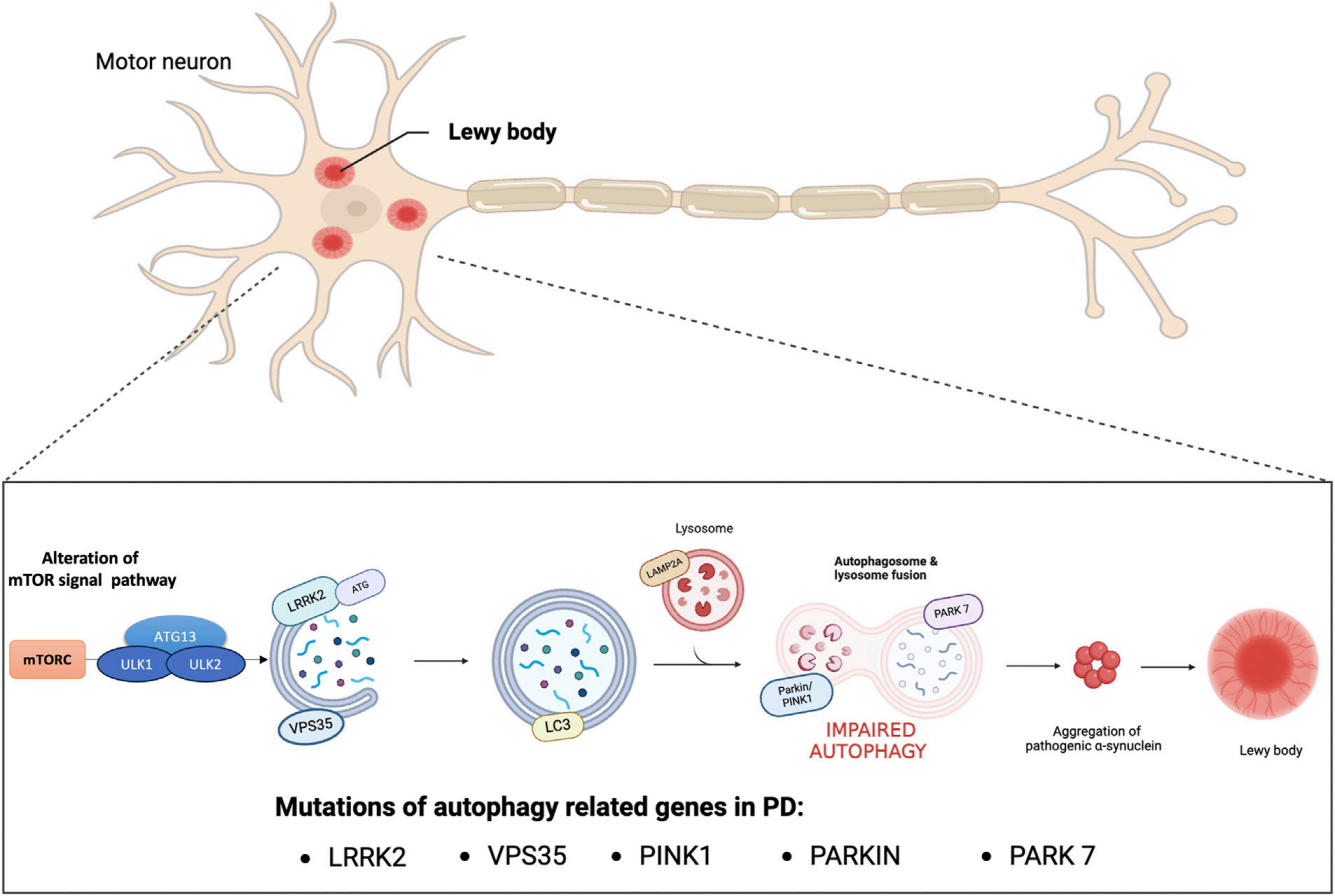
Impaired autophagy in PD. Genetic mutations and encoded proteins related to PD are functionally linked to the autophagy pathway. Disease-associated mutations impair this essential degradation process. Autophagy deficiency or blockage of autophagic flux increases the oligomerization and aggregation of α-synuclein proteins forming Lewy bodies which contributes to neurodegeneration.

PD has been linked to aging, environmental toxins, and genetic mutation. In fact, the molecular pathogenesis of this disease includes various factors, such as oxidative stress, impairment of synaptic transmission, neuroinflammation, mitochondrial dysfunction and impairment of protein homeostasis (Trist et al., 2019). The alteration of protein homeostasis and the accumulation of damaged or abnormally modified proteins characteristic of PD are closely related to the impairment of the autophagic mechanism (Cerri and Blandini, 2019). Autophagy is a catabolic cellular process responsible for the degradation and recycling of cellular components therefore, it plays a crucial role in maintaining cellular homeostasis by removing damaged proteins and organelles (Parzych and Klionsky, 2014). Research suggests that dysfunctions in autophagy mechanisms can lead to the accumulation of harmful substances such as misfolded proteins or damaged mitochondria, thus contributing to the development and neurodegeneration of PD (Han et al., 2019). Physiologically, thanks to the autophagic mechanism, misfolded proteins and damaged organelles are transported to the lysosome for degradation and once inside the lumen of the lysosome are hydrolyzed by a series of proteases, known as cathepsins (Sorice, 2022). In particular, autophagy pathways include macroautophagy, chaperone-mediated autophagy (CMA) and microautophagy, and involve genes and proteins related to autophagy (ATGs). Macroautophagy occurs under normal conditions of cell growth and differentiation and one of its main roles is to maintain proper cellular homeostasis. Furthermore, under stressful conditions such as starvation or energy deficit the autophagic process could be induced. During this process, unwanted cytosolic contents are delivered to degradative organelles, such as lysosomes and vacuoles, that control their digestion and final elimination. In particular, dysfunctional organelles are isolated by a phagophore that forms a double-membrane vesicle called an autophagosome. At this stage, the ATGs that initiate autophagosome formation are the unc-51-like protein complex that activates autophagy kinase 1 and 2 (ULK1/ULK2) regulated by subsequent phosphorylation reactions. The activated ULK1 complex phosphorylates the proteins Beclin1 and Ambra1 to form the active vacuolar protein sorting 34 (Vps34) complex (Sun, 2016) which consequently recruits the autophagy proteins ATG12 and ATG5 for phagophore formation. Once the autophagosome is formed light chain 3 (LC3) allows binding to autophagic substrates and/or proteins that mediate cargo selectivity (Galluzzi et al., 2017). Finally, the autophagosome fuses with the lysosome to form the autophagic vacuole and initiate degradation of the cytosolic cargo. The degraded luminal contents are returned to the cytosol to be recycled through various metabolic reactions (Levine and Klionsky, 2004). Otherwise, microautophagy recycles small portions of cytoplasm that are directly enveloped by the lysosome (Li et al., 2012). Finally, CMA selectively degrades proteins containing the KFERQ amino acid sequence that are transported to the lysosome through the combined actions of lysosomal associated membrane protein 2 (LAMP2A) and heat shock chaperone protein 70 (HSC70). One of the proteins with central regulatory activity of the autophagic process is the mammalian target of rapamycin complex 1 (mTORC1). Through direct binding to the ULK1 and ULK2 complexes, active mTORC1 constitutes a potent repressor of autophagy. For example, under nutrient starvation conditions, phosphorylation of ULK1/ULK2 by mTOR is reduced, and adenosine monophosphate (AMP)-activated protein kinase (AMPK) phosphorylates the ULK1/ULK2 protein complex at a site different from that of mTORC1 with resulting in induction of autophagy. Otherwise, in the presence of high levels of nutrients mTORC1 is active and inhibits autophagy, therefore the correct balance of mTOR signaling under physiological conditions is important to ensure normal cellular health (Dibble and Cantley, 2015). In this regard, studies have demonstrated an alteration of mTOR signaling in PD where there is an overactivation of mTOR which in turn reduces neuronal autophagy and promotes the accumulation of α-syn (Ghavami et al., 2014; Recasens et al., 2014; Gao et al., 2015; Wong and Krainc, 2017; Zhang Z. et al., 2021). In fact, the abnormal aggregation of proteins and the deficits of degradation processes misfolded proteins are the most common pathological signs of neurodegenerative diseases such as PD and Alzheimer’s disease (AD) (Filippone et al., 2022). In AD, defects in the macro-autophagy pathway due to genetic mutations, environmental factors, or aging may contribute to the accumulation of abnormal protein aggregates. Mutations in presenilin-1 alter the pH of the lysosome, thus decreasing the activity of autophagy which can increase the accumulation of Aβ peptides promoting neurodegeneration (Sharoar et al., 2021). Furthermore, in AD there is a defect in the mitophagy process with consequent accumulation of autophagic vacuoles which increase oxidative stress and reduction of mitochondria which leads to a reduction in ATP production. These events cause less degradation of Aβ peptides whose accumulation blocks the proteins that activate mitophagy, thus establishing a vicious circle that further increases neurodegeneration processes (Fang et al., 2019; Bera et al., 2020). Furthermore, also in other neurodegenerative diseases such as in polyglutamine diseases such as Huntington’s disease (HD), spinal and bulbar muscular atrophy, dentatorubral-pallidoluisian atrophy, spinocerebellar ataxia (SCA) and spinocerebellar ataxia (SCA) the alteration of autophagic processes due to genetic mutations are responsible for the formation of toxic oligomers and aggregates (Fu et al., 2017; Fujikake et al., 2018). Concerning the PD, once the autophagic pathway is compromised, the clearance of proteins such as α-synuclein fails (Guo et al., 2018). Numerous studies have demonstrated that α-synuclein positive Lewy bodies in the substantia nigra of the PD brain showed immunoreactivity for LC3 confirming the role of autophagy in the pathogenesis of PD (Mamais et al., 2018; Minakaki et al., 2018; Tu et al., 2021; Oh et al., 2022). Furthermore, a large group of genes associated with familial and sporadic PD are strongly linked to macroautophagy, CMA and downstream lysosomal function, such as mutations in SNCA, leucine-rich repeat kinase 2 (LRRK2–PARK8), vacuolar protein sorting 35 (VPS35), Parkin RBR E3 ubiquitin-protein ligase (PARKIN), PTEN-induced kinase 1 (PINK1) and Parkinsonism associated deglycase (PARK7) (Nalls et al., 2019). Regarding alterations of genes involved in macroautophagy, numerous studies showed that mutations in LRRK2 are the main cause of hereditary PD (Kluss et al., 2019). In particular, mutation of LRRK2 at the G20195 site disrupts the transport of autophagic vesicles into cortical neurons, indicating defective trafficking of neuronal autophagosomes resulting in accumulation of alpha-synuclein. Furthermore, a recent study showed that LRRK2 exacerbates cytotoxicity by disrupting the autophagic clearance of accumulated protein aggregates (Hu et al., 2023). Besides its role in macroautophagy, few studies have reported effects of LRRK2 on CMA. Under normal conditions, wild-type LRRK2 can be degraded by CMA. However, pathogenic mutants that overexpress wild-type LRRK2 can block CMA by inhibiting the lysosomal membrane translocation complex. As a result, there is accumulation of other CMA substrates, including α-synuclein (Ho et al., 2020). In addition to the evidence mentioned above, the pathogenic role of autophagic dysfunction in PD has been related to mutations in other genes such as homozygous or heterozygous mutations in PINK1 and in PARKIN (Quinn et al., 2020). These mutations have been identified as the most common causes of early-onset autosomal recessive PD, with overall frequencies of 1%–9% for PINK1 depending on ethnicity and nearly 50% for PRKN in young patients with PD (≤40 years) (Lucking et al., 2000; Puschmann, 2013). Both proteins regulate autophagy for the selective degradation of damaged mitochondria. PINK1 or PRKN mutations result in impaired mitophagic function and may contribute to selective neurodegeneration in the substantia nigra (Figure 1) (Sliter et al., 2018). Therefore, promotion of autophagy is presumed to exert a protective effect in PD. Elimination of Lewy bodies through activation of the autophagy pathway could prevent toxicity, thus reducing the progression of PD (Suresh et al., 2020). Researchers are actively investigating various approaches, including small molecules capable of increasing α-syn clearance as mTOR inhibitor (including CCI-779 known as Temsirolimus; and AP23573, known as Ridaforolimus), gene therapies, and lifestyle interventions, to target autophagy as a potential therapeutic avenue for PD (Zhang K. et al., 2021). Furthermore, departing from the classic vision of autophagy as a mechanism of proteostasis through protein degradation, recent scientific evidence has discovered that autophagy also presents non-canonical functions that concern the regulation of unconventional secretory processes (Rabouille et al., 2012). In particular, a new non-degradative role of autophagy has emerged, leading to the notion of secretory autophagy. Proteins with the amino-terminal signal peptide are secreted by exocytosis following a well-defined secretory pathway through the endoplasmic reticulum and the Golgi apparatus. However, some cytosolic proteins lack these signal peptides and are unable to enter the endoplasmic reticulum. These cytosolic proteins are secreted by several unconventional processes including secretory autophagy (New and Thomas, 2019; Gonzalez et al., 2020). Secretory autophagy is becoming of increasing importance to explain the secretion of a number of peptides that have critical biological importance. It has recently been demonstrated that secretory autophagy could play a role in the release of proteins subject to aggregation, thus highlighting a new pathophysiological mechanism of some neurodegenerative diseases such as PD and AD (Ejlerskov et al., 2013). Increasing evidence indicates a relevant role for secretory autophagy in the pathological secretion of α-synuclein protein aggregates and amyloid beta peptide as cellular response mechanisms to degenerative diseases [(Ejlerskov et al., 2013; Nilsson and Saido, 2014)]. Furthermore, secretion of the PD-associated Park7/DJ-1 protein is mediated by secretory autophagy. Park7 secretion is induced by autophagy through activation of AMPK and ULK1 and is suppressed in animals deficient in Atg5, Atg9, or Atg16lL (Urano et al., 2018). However, the potential implications of secretory autophagy in neurodegenerative diseases are still unclear. Therefore, further research is needed to better understand the role of secretory autophagy as a pathophysiological mechanism and as a potential therapeutic target.

## 4 How gender differences affect autophagic processes in PD

Gender differences exist in various diseases and may affect not only the disease itself, but also drug therapy. Recent studies suggest that sex differences influence autophagy processes in healthy states as well as in disease (Shang et al., 2021). Several X-linked genes, such as ATP6AP2 (ATPase H+ transporting accessory protein 2), influence autophagic processes and mutations in these genes can cause human disease. Specifically, ATP6AP2 appears to be a gene associated with Parkinson’s disease, in particular X-linked parkinsonism with spasticity/XPDS. Loss-of-function mutations in ATP6AP2 lead to accumulation of autophagosomes and defects in lysosomal clearance. To confirm this, similar neurodegeneration and cognitive impairment were observed in a study with male ATP6AP2 knockout (KO) mice; however, female KO mouse models are needed to confirm sex differences in autophagy (Dubos et al., 2015). Therefore, understanding the biology of sex differences in autophagy is necessary (Lista et al., 2011). Gender differences in autophagy in the context of PD represent an interesting and complex aspect of neuroscientific research. Studies showed that the regulation of autophagy may be influenced by gender factors, with differential expression of key autophagy-related genes between the sexes. These variations may be reflected in the dynamics of aggregated protein elimination and cell clearance, processes that are critical for neuronal health. In addition, the response to autophagy activation may differ, leading to a variety of clinical presentations of the disease between men and women. Recent studies have shown gender differences in autophagy processes in the cerebral cortex. De Miranda et al. (2019) suggest that the female brain could upregulate mitophagy, avoiding neuronal cell death. In fact, in their study, they observed a greater presence of LC3II autophagosome markers in male control animals compared to females, suggesting a higher amount of constitutive autophagy in male brain. Following brain injury, autophagosomes increased in female, reaching levels in male control animals; consequently, Demarest et al. (2016) hypothesize that the induction of autophagy/mitophagy represents a sex-specific compensatory mechanism to protect from mitochondrial dysfunction, prevent the accumulation of damaged cellular components and reduce cell death. De Miranda et al. (2019), studying rotenone-induced dopaminergic neurodegeneration in adult male and female rats, confirmed this evidence by demonstrating an accumulation of the autophagy receptor protein p62 (also called sequestosome 1, SQSTM1) and a decrease in lysosomal associated membrane protein 1 (Lamp1) in male rats following rotenone treatment suggesting a defect in autophagic flux, which might contribute to increased levels of a-synuclein. In contrast, female rats showed an increase of p62-SQSTM1 levels, but did not show a decrease in Lamp1, indicating an upregulation of autophagic flux and consequently, a reduced accumulation of a-synuclein. Studies in the literature showed that among the many genes involved in autophagic processes, LRRK2 and ATG appear to be the most affected by gender difference.

### 4.1 LRRK2 role

The involvement of LRRK2 in Parkinson’s disease appears to have significant differences between men and women. Recent studies suggest that men who carry LRRK2 mutations are more likely to develop PD, while women may face faster disease progression once affected and experience more severe symptoms, despite the lower incidence of such mutations. This intricate and complex gender variation could be due to a combination of genetic, hormonal, and environmental factors that interact uniquely in LRRK2-related disease processes. Some studies have observed a female preponderance in PD associated with LRRK2 (Cilia et al., 2014; San Luciano et al., 2017), while other studies found no gender differences (Alcalay et al., 2013; Gan-Or et al., 2015). A higher female prevalence of the LRRK2 mutation suggests a role of gender-related risk factors in Parkinson’s patients, particularly those carrying the G2019S mutation. G2019S, the most common mutation, is responsible for 1% of sporadic and 4% of familial PD. In contrast, no gender difference was observed in the prevalence of patients carrying the G2385R mutation. Given the unclear gender difference in the prevalence of LRRK2-associated Parkinson’s disease, Chen et al. (2020) conducted a meta-analysis to identify the hypothesis of the gender effect in LRRK2-associated Parkinson’s disease, confirming a higher prevalence in female patients carrying LRRK2 variants, particularly the G2019S mutation. Confirming this, Cilia et al. (2014) showed that PD patients with LRRK2 mutations are more likely to be women, suggesting a higher genetic burden.

### 4.2 ATG role

Autophagic processes take place through the activity of multiple ATGs. Currently, 31 ATG genes have been identified, each of which plays a key role in different stages of autophagy. In particular, deficits in key autophagy genes, such as autophagy-related gene 5 (ATG5) and autophagy-related gene 7 (ATG7), have been found to lead to neurodegeneration and protein accumulation in the remaining neurons (Hara et al., 2006; Komatsu et al., 2006; Zhang et al., 2018). Although ATG plays a central role in the regulation of autophagy, its involvement in PD appears to differ between men and women, opening a research perspective that reflects the complexity of the pathogenesis of this neurodegenerative condition (Lynch-Day et al., 2012). As a result, research is focusing on how gender differences affect ATG gene expression and how this is reflected in individual susceptibility, clinical presentation, and disease progression. ATG5 plays a key role in autophagosome formation and recent studies have shown a correlation between abnormal expression or gene deletion of ATG5 and the onset of several neurodegenerative diseases. This evidence suggests that ATG5 gene expression and polymorphism influence the development and progression of PD, however, the specific correlation between ATG5 and PD susceptibility is not yet fully known (Han et al., 2022). Sepulveda et al. in their study showed that ATG5 expression was more correlated with male PD patients, potentially contributing to the higher prevalence of PD in males (Sepulveda et al., 2022). ATG7 also plays a key role in autophagic processes, and increasing evidence suggests that ATG7 may be implicated in the pathogenesis of PD (Zhao et al., 2020). Chen et al. (2013) demonstrated that variations in the expression level of ATG7 gene may contribute to PD pathogenesis by altering autophagic activity. To date, however, there is no significant evidence demonstrating substantial differences in the role of ATG7 between men and women with Parkinson’s.

## 5 Discussion

PD is the most widespread neurodegenerative disease characterized by a gradual damage of dopaminergic neurons in the substantia nigra pars compacta and presence of α-synuclein (α-syn)-rich cytoplasmic neuronal inclusion named Lewy bodies (Poewe et al., 2017). In this review, a detailed analysis was carried out to summarize gender differences in autophagic processes associates with PD. Based on the studies cited, autophagy, involved in the etiopathogenesis of PD, may present significant variations depending on sex. Promotion of autophagy is known to exert a protective effect in Parkinson’s disease. Elimination of Lewy bodies through activation of the autophagic pathway could prevent toxicity, thus reducing the progression of Parkinson’s disease. Studies have shown that the regulation of autophagy can be influenced by gender factors, with differential expression of key autophagy-related genes between the sexes. Indeed, while some evidence suggests greater autophagic activation in men with Parkinson’s, women may have distinct regulatory patterns. These variations may be reflected in the dynamics of aggregated protein and cellular clearance, all processes critical for neuronal health. Therefore, the different activation of autophagy may lead to a variety of clinical presentations of the disease between men and women. The existing literature on gender differences in autophagic processes associated with Parkinson’s disease, shown that the main proteins involved are ATG and LRRK2. ATGs play a pivotal role across multiple stages of the autophagic pathway. Current analyses revealed gender differences specifically within ATG5 gene, while findings regarding ATG7 reveal no such gender variation. On the other hand, investigations into LRRK2 have illuminated substantial differences between males and females, showing a higher prevalence of its mutations among women. In conclusion, the analysis of gender differences in autophagic processes associated with Parkinson’s reveals a complex and intriguing panorama. The studies reviewed demonstrated significant differences in autophagic regulatory mechanisms between men and women with Parkinson’s, suggesting that such gender differences significantly influence disease progression and manifestation. Therefore, exploring into gender differences in autophagic processes will not only allow for a better understanding of the biology underlying PD, but could significantly contribute to improving the quality of life of Parkinson’s patients through more targeted and personalized treatments. Indeed, a gender-based targeted therapy for Parkinson’s disease could offer several significant advantages, and recognition of the role of autophagy in the context of gender differences in PD could offer new opportunities for innovative research and therapeutic development.

## References

[B1] Abraham D. S. Gruber-Baldini A. L. Magder L. S. McArdle P. F. Tom S. E. Barr E. (2019). Sex differences in Parkinson's disease presentation and progression. Park. Relat. Disord. 69, 48–54. 10.1016/j.parkreldis.2019.10.019 PMC6982644 31677455

[B2] Alcalay R. N. Mirelman A. Saunders-Pullman R. Tang M. X. Mejia Santana H. Raymond D. (2013). Parkinson disease phenotype in Ashkenazi Jews with and without LRRK2 G2019S mutations. Mov. Disord. 28 (14), 1966–1971. 10.1002/mds.25647 24243757 PMC3859844

[B3] Augustine E. F. Perez A. Dhall R. Umeh C. C. Videnovic A. Cambi F. (2015). Sex differences in clinical features of early, treated Parkinson's disease. PLoS One 10 (7), e0133002. 10.1371/journal.pone.0133002 26171861 PMC4501841

[B4] Bera S. Camblor-Perujo S. Calleja Barca E. Negrete-Hurtado A. Racho J. De Bruyckere E. (2020). AP-2 reduces amyloidogenesis by promoting BACE1 trafficking and degradation in neurons. EMBO Rep. 21 (6), e47954. 10.15252/embr.201947954 32323475 PMC7271323

[B5] Bianco A. Antonacci Y. Liguori M. (2023). Sex and gender differences in neurodegenerative diseases: challenges for therapeutic opportunities. Int. J. Mol. Sci. 24 (7), 6354. 10.3390/ijms24076354 37047320 PMC10093984

[B6] Blauwendraat C. Iwaki H. Makarious M. B. Bandres-Ciga S. Leonard H. L. Grenn F. P. (2021). Investigation of autosomal genetic sex differences in Parkinson's disease. Ann. Neurol. 90 (1), 35–42. 10.1002/ana.26090 33901317 PMC8422907

[B7] Bourque M. Morissette M. Soulet D. Di Paolo T. (2023). Impact of sex on neuroimmune contributions to Parkinson's disease. Brain Res. Bull. 199, 110668. 10.1016/j.brainresbull.2023.110668 37196734

[B8] Brann D. W. Dhandapani K. Wakade C. Mahesh V. B. Khan M. M. (2007). Neurotrophic and neuroprotective actions of estrogen: basic mechanisms and clinical implications. Steroids 72 (5), 381–405. 10.1016/j.steroids.2007.02.003 17379265 PMC2048656

[B9] Cai J. Kim J. L. Wang Y. Baumeister T. R. Zhu M. Liu A. (2023). Sex, myelin, and clinical characteristics of Parkinson's disease. Front. Neurosci. 17, 1235524. 10.3389/fnins.2023.1235524 37781247 PMC10535348

[B10] Cerri S. Blandini F. (2019). Role of autophagy in Parkinson's disease. Curr. Med. Chem. 26 (20), 3702–3718. 10.2174/0929867325666180226094351 29484979

[B11] Cerri S. Mus L. Blandini F. (2019). Parkinson's disease in women and men: what's the difference? J. Park. Dis. 9 (3), 501–515. 10.3233/JPD-191683 PMC6700650 31282427

[B12] Chen D. Pang S. Feng X. Huang W. Hawley R. G. Yan B. (2013). Genetic analysis of the ATG7 gene promoter in sporadic Parkinson's disease. Neurosci. Lett. 534, 193–198. 10.1016/j.neulet.2012.12.039 23295909

[B13] Chen W. Yan X. Lv H. Liu Y. He Z. Luo X. (2020). Gender differences in prevalence of LRRK2-associated Parkinson disease: a meta-analysis of observational studies. Neurosci. Lett. 715, 134609. 10.1016/j.neulet.2019.134609 31698024

[B14] Cilia R. Siri C. Rusconi D. Allegra R. Ghiglietti A. Sacilotto G. (2014). LRRK2 mutations in Parkinson's disease: confirmation of a gender effect in the Italian population. Park. Relat. Disord. 20 (8), 911–914. 10.1016/j.parkreldis.2014.04.016 PMC4144811 24816003

[B15] Dahodwala N. Pei Q. Schmidt P. (2016). Sex differences in the clinical progression of Parkinson's disease. J. Obstet. Gynecol. Neonatal Nurs. 45 (5), 749–756. 10.1016/j.jogn.2016.05.002 PMC5021611 27444842

[B16] Demarest T. G. Waite E. L. Kristian T. Puche A. C. Waddell J. McKenna M. C. (2016). Sex-dependent mitophagy and neuronal death following rat neonatal hypoxia-ischemia. Neuroscience 335, 103–113. 10.1016/j.neuroscience.2016.08.026 27555552 PMC5580242

[B17] De Miranda B. R. Fazzari M. Rocha E. M. Castro S. Greenamyre J. T. (2019). Sex differences in rotenone sensitivity reflect the male-to-female ratio in human Parkinson's disease incidence. Toxicol. Sci. 170 (1), 133–143. 10.1093/toxsci/kfz082 30907971 PMC6592185

[B18] Dibble C. C. Cantley L. C. (2015). Regulation of mTORC1 by PI3K signaling. Trends Cell. Biol. 25 (9), 545–555. 10.1016/j.tcb.2015.06.002 26159692 PMC4734635

[B19] Dluzen D. E. (2000). Neuroprotective effects of estrogen upon the nigrostriatal dopaminergic system. J. Neurocytol. 29 (5-6), 387–399. 10.1023/a:1007117424491 11424955

[B20] Dubos A. Castells-Nobau A. Meziane H. Oortveld M. A. Houbaert X. Iacono G. (2015). Conditional depletion of intellectual disability and Parkinsonism candidate gene ATP6AP2 in fly and mouse induces cognitive impairment and neurodegeneration. Hum. Mol. Genet. 24 (23), 6736–6755. 10.1093/hmg/ddv380 26376863 PMC4634377

[B21] Ejlerskov P. Rasmussen I. Nielsen T. T. Bergström A. L. Tohyama Y. Jensen P. H. (2013). Tubulin polymerization-promoting protein (TPPP/p25α) promotes unconventional secretion of α-synuclein through exophagy by impairing autophagosome-lysosome fusion. J. Biol. Chem. 288 (24), 17313–17335. 10.1074/jbc.M112.401174 23629650 PMC3682534

[B22] Fang E. F. Hou Y. Palikaras K. Adriaanse B. A. Kerr J. S. Yang B. (2019). Mitophagy inhibits amyloid-β and tau pathology and reverses cognitive deficits in models of Alzheimer's disease. Nat. Neurosci. 22 (3), 401–412. 10.1038/s41593-018-0332-9 30742114 PMC6693625

[B23] Filippone A. Esposito E. Mannino D. Lyssenko N. Praticò D. (2022). The contribution of altered neuronal autophagy to neurodegeneration. Pharmacol. Ther. 238, 108178. 10.1016/j.pharmthera.2022.108178 35351465 PMC9510148

[B24] Fu Y. Wu P. Pan Y. Sun X. Yang H. Difiglia M. (2017). A toxic mutant huntingtin species is resistant to selective autophagy. Nat. Chem. Biol. 13 (11), 1152–1154. 10.1038/nchembio.2461 28869595

[B25] Fujikake N. Shin M. Shimizu S. (2018). Association between autophagy and neurodegenerative diseases. Front. Neurosci. 12, 255. 10.3389/fnins.2018.00255 29872373 PMC5972210

[B26] Galluzzi L. Baehrecke E. H. Ballabio A. Boya P. Bravo-San Pedro J. M. Cecconi F. (2017). Molecular definitions of autophagy and related processes. EMBO J. 36 (13), 1811–1836. 10.15252/embj.201796697 28596378 PMC5494474

[B27] Gan-Or Z. Leblond C. S. Mallett V. Orr-Urtreger A. Dion P. A. Rouleau G. A. (2015). LRRK2 mutations in Parkinson disease; a sex effect or lack thereof? A meta-analysis. Park. Relat. Disord. 21 (7), 778–782. 10.1016/j.parkreldis.2015.05.002 25962553

[B28] Gao S. Duan C. Gao G. Wang X. Yang H. (2015). Alpha-synuclein overexpression negatively regulates insulin receptor substrate 1 by activating mTORC1/S6K1 signaling. Int. J. Biochem. Cell. Biol. 64, 25–33. 10.1016/j.biocel.2015.03.006 25813876

[B29] Georgiev D. Hamberg K. Hariz M. Forsgren L. Hariz G. M. (2017). Gender differences in Parkinson's disease: a clinical perspective. Acta Neurol. Scand. 136 (6), 570–584. 10.1111/ane.12796 28670681

[B30] Ghavami S. Shojaei S. Yeganeh B. Ande S. R. Jangamreddy J. R. Mehrpour M. (2014). Autophagy and apoptosis dysfunction in neurodegenerative disorders. Prog. Neurobiol. 112, 24–49. 10.1016/j.pneurobio.2013.10.004 24211851

[B31] Gillies G. E. McArthur S. (2010). Estrogen actions in the brain and the basis for differential action in men and women: a case for sex-specific medicines. Pharmacol. Rev. 62 (2), 155–198. 10.1124/pr.109.002071 20392807 PMC2879914

[B32] Gillies G. E. Murray H. E. Dexter D. McArthur S. (2004). Sex dimorphisms in the neuroprotective effects of estrogen in an animal model of Parkinson's disease. Pharmacol. Biochem. Behav. 78 (3), 513–522. 10.1016/j.pbb.2004.04.022 15251260

[B33] Gonzalez C. D. Resnik R. Vaccaro M. I. (2020). Secretory autophagy and its relevance in metabolic and degenerative disease. Front. Endocrinol. (Lausanne) 11, 266. 10.3389/fendo.2020.00266 32477265 PMC7232537

[B34] Guo F. Liu X. Cai H. Le W. (2018). Autophagy in neurodegenerative diseases: pathogenesis and therapy. Brain Pathol. 28 (1), 3–13. 10.1111/bpa.12545 28703923 PMC5739982

[B35] Haaxma C. A. Bloem B. R. Borm G. F. Oyen W. J. Leenders K. L. Eshuis S. (2007). Gender differences in Parkinson's disease. J. Neurol. Neurosurg. Psychiatry 78 (8), 819–824. 10.1136/jnnp.2006.103788 17098842 PMC2117736

[B36] Han J. Feng G. Wu J. Zhang Y. Long Z. Yao X. (2022). Association of ATG5 gene polymorphism with Parkinson's disease in a Han Chinese population. Acta Neurol. Belg 122 (4), 1049–1056. 10.1007/s13760-021-01814-y 34661876 PMC9300489

[B37] Han X. Sun S. Sun Y. Song Q. Zhu J. Song N. (2019). Small molecule-driven NLRP3 inflammation inhibition via interplay between ubiquitination and autophagy: implications for Parkinson disease. Autophagy 15 (11), 1860–1881. 10.1080/15548627.2019.1596481 30966861 PMC6844502

[B38] Hara T. Nakamura K. Matsui M. Yamamoto A. Nakahara Y. Suzuki-Migishima R. (2006). Suppression of basal autophagy in neural cells causes neurodegenerative disease in mice. Nature 441 (7095), 885–889. 10.1038/nature04724 16625204

[B39] Ho P. W. Leung C. T. Liu H. Pang S. Y. Lam C. S. Xian J. (2020). Age-dependent accumulation of oligomeric SNCA/α-synuclein from impaired degradation in mutant LRRK2 knockin mouse model of Parkinson disease: role for therapeutic activation of chaperone-mediated autophagy (CMA). Autophagy 16 (2), 347–370. 10.1080/15548627.2019.1603545 30983487 PMC6984454

[B40] Hu J. Zhang D. Tian K. Ren C. Li H. Lin C. (2023). Small-molecule LRRK2 inhibitors for PD therapy: current achievements and future perspectives. Eur. J. Med. Chem. 256, 115475. 10.1016/j.ejmech.2023.115475 37201428

[B41] Jurado-Coronel J. C. Cabezas R. Avila Rodriguez M. F. Echeverria V. Garcia-Segura L. M. Barreto G. E. (2018). Sex differences in Parkinson's disease: features on clinical symptoms, treatment outcome, sexual hormones and genetics. Front. Neuroendocrinol. 50, 18–30. 10.1016/j.yfrne.2017.09.002 28974386

[B42] Kluss J. H. Mamais A. Cookson M. R. (2019). LRRK2 links genetic and sporadic Parkinson's disease. Biochem. Soc. Trans. 47 (2), 651–661. 10.1042/BST20180462 30837320 PMC6563926

[B43] Komatsu M. Waguri S. Chiba T. Murata S. Iwata J. Tanida I. (2006). Loss of autophagy in the central nervous system causes neurodegeneration in mice. Nature 441 (7095), 880–884. 10.1038/nature04723 16625205

[B44] Kompoliti K. Adler C. H. Raman R. Pincus J. H. Leibowitz M. T. Ferry J. J. (2002). Gender and pramipexole effects on levodopa pharmacokinetics and pharmacodynamics. Neurology 58 (9), 1418–1422. 10.1212/wnl.58.9.1418 12011296

[B45] Kumagai T. Nagayama H. Ota T. Nishiyama Y. Mishina M. Ueda M. (2014). Sex differences in the pharmacokinetics of levodopa in elderly patients with Parkinson disease. Clin. Neuropharmacol. 37 (6), 173–176. 10.1097/WNF.0000000000000051 25384078

[B46] Levine B. Klionsky D. J. (2004). Development by self-digestion: molecular mechanisms and biological functions of autophagy. Dev. Cell. 6 (4), 463–477. 10.1016/s1534-5807(04)00099-1 15068787

[B47] Li W. W. Li J. Bao J. K. (2012). Microautophagy: lesser-known self-eating. Cell. Mol. Life Sci. 69 (7), 1125–1136. 10.1007/s00018-011-0865-5 22080117 PMC11114512

[B48] Lista P. Straface E. Brunelleschi S. Franconi F. Malorni W. (2011). On the role of autophagy in human diseases: a gender perspective. J. Cell. Mol. Med. 15 (7), 1443–1457. 10.1111/j.1582-4934.2011.01293.x 21362130 PMC3823190

[B49] Liu B. Dluzen D. E. (2007). Oestrogen and nigrostriatal dopaminergic neurodegeneration: animal models and clinical reports of Parkinson's disease. Clin. Exp. Pharmacol. Physiol. 34 (7), 555–565. 10.1111/j.1440-1681.2007.04616.x 17581209

[B50] Lubomski M. Louise R. R. Lee W. Bertram K. L. Williams D. R. (2014). Sex differences in Parkinson's disease. J. Clin. Neurosci. 21 (9), 1503–1506. 10.1016/j.jocn.2013.12.016 24767694

[B51] Lucking C. B. Durr A. Bonifati V. Vaughan J. De Michele G. Gasser T. (2000). Association between early-onset Parkinson's disease and mutations in the parkin gene. N. Engl. J. Med. 342 (21), 1560–1567. 10.1056/NEJM200005253422103 10824074

[B52] Lynch-Day M. A. Mao K. Wang K. Zhao M. Klionsky D. J. (2012). The role of autophagy in Parkinson's disease. Cold Spring Harb. Perspect. Med. 2 (4), a009357. 10.1101/cshperspect.a009357 22474616 PMC3312403

[B53] Mamais A. Manzoni C. Nazish I. Arber C. Sonustun B. Wray S. (2018). Analysis of macroautophagy related proteins in G2019S LRRK2 Parkinson's disease brains with Lewy body pathology. Brain Res. 1701, 75–84. 10.1016/j.brainres.2018.07.023 30055128 PMC6361106

[B54] Martinelli P. Contin M. Scaglione C. Riva R. Albani F. Baruzzi A. (2003). Levodopa pharmacokinetics and dyskinesias: are there sex-related differences? Neurol. Sci. 24 (3), 192–193. 10.1007/s10072-003-0125-z 14598082

[B55] Minakaki G. Menges S. Kittel A. Emmanouilidou E. Schaeffner I. Barkovits K. (2018). Autophagy inhibition promotes SNCA/alpha-synuclein release and transfer via extracellular vesicles with a hybrid autophagosome-exosome-like phenotype. Autophagy 14 (1), 98–119. 10.1080/15548627.2017.1395992 29198173 PMC5846507

[B56] Nalls M. A. Blauwendraat C. Vallerga C. L. Heilbron K. Bandres-Ciga S. Chang D. (2019). Identification of novel risk loci, causal insights, and heritable risk for Parkinson's disease: a meta-analysis of genome-wide association studies. Lancet Neurol. 18 (12), 1091–1102. 10.1016/S1474-4422(19)30320-5 31701892 PMC8422160

[B57] Nechushtai L. Frenkel D. Pinkas-Kramarski R. (2023). Autophagy in Parkinson's disease. Biomolecules 13 (10), 1435. 10.3390/biom13101435 37892117 PMC10604695

[B58] New J. Thomas S. M. (2019). Autophagy-dependent secretion: mechanism, factors secreted, and disease implications. Autophagy 15 (10), 1682–1693. 10.1080/15548627.2019.1596479 30894055 PMC6735501

[B59] Nilsson P. Saido T. C. (2014). Dual roles for autophagy: degradation and secretion of Alzheimer's disease Aβ peptide. Bioessays 36 (6), 570–578. 10.1002/bies.201400002 24711225 PMC4316186

[B60] Oh C. K. Dolatabadi N. Cieplak P. Diaz-Meco M. T. Moscat J. Nolan J. P. (2022). S-nitrosylation of p62 inhibits autophagic flux to promote α-synuclein secretion and spread in Parkinson's disease and Lewy body dementia. J. Neurosci. 42 (14), 3011–3024. 10.1523/JNEUROSCI.1508-21.2022 35169022 PMC8985870

[B61] Parzych K. R. Klionsky D. J. (2014). An overview of autophagy: morphology, mechanism, and regulation. Antioxid. Redox Signal 20 (3), 460–473. 10.1089/ars.2013.5371 23725295 PMC3894687

[B62] Poewe W. Seppi K. Tanner C. M. Halliday G. M. Brundin P. Volkmann J. (2017). Parkinson disease. Nat. Rev. Dis. Prim. 3 (1), 17013–17021. 10.1038/nrdp.2017.13 28332488

[B63] Puschmann A. (2013). Monogenic Parkinson's disease and parkinsonism: clinical phenotypes and frequencies of known mutations. Park. Relat. Disord. 19 (4), 407–415. 10.1016/j.parkreldis.2013.01.020 23462481

[B64] Quinn P. M. J. Moreira P. I. Ambrosio A. F. Alves C. H. (2020). PINK1/PARKIN signalling in neurodegeneration and neuroinflammation. Acta Neuropathol. Commun. 8 (1), 189. 10.1186/s40478-020-01062-w 33168089 PMC7654589

[B65] Rabouille C. Malhotra V. Nickel W. (2012). Diversity in unconventional protein secretion. J. Cell. Sci. 125 (Pt 22), 5251–5255. 10.1242/jcs.103630 23377655

[B66] Ramesh S. Arachchige A. (2023). Depletion of dopamine in Parkinson's disease and relevant therapeutic options: a review of the literature. AIMS Neurosci. 10 (3), 200–231. 10.3934/Neuroscience.2023017 37841347 PMC10567584

[B67] Recasens A. Dehay B. Bove J. Carballo-Carbajal I. Dovero S. Perez-Villalba A. (2014). Lewy body extracts from Parkinson disease brains trigger α-synuclein pathology and neurodegeneration in mice and monkeys. Ann. Neurol. 75 (3), 351–362. 10.1002/ana.24066 24243558

[B68] Reekes T. H. Higginson C. I. Ledbetter C. R. Sathivadivel N. Zweig R. M. Disbrow E. A. (2020). Sex specific cognitive differences in Parkinson disease. NPJ Park. Dis. 6, 7. 10.1038/s41531-020-0109-1 PMC7142103 32284961

[B69] San Luciano M. Wang C. Ortega R. A. Giladi N. Marder K. Bressman S. (2017). Sex differences in LRRK2 G2019S and idiopathic Parkinson's Disease. Ann. Clin. Transl. Neurol. 4 (11), 801–810. 10.1002/acn3.489 29159192 PMC5682117

[B70] Sepulveda D. Grunenwald F. Vidal A. Troncoso-Escudero P. Cisternas-Olmedo M. Villagra R. (2022). Insulin-like growth factor 2 and autophagy gene expression alteration arise as potential biomarkers in Parkinson's disease. Sci. Rep. 12 (1), 2038. 10.1038/s41598-022-05941-1 35132125 PMC8821705

[B71] Shang D. Wang L. Klionsky D. J. Cheng H. Zhou R. (2021). Sex differences in autophagy-mediated diseases: toward precision medicine. Autophagy 17 (5), 1065–1076. 10.1080/15548627.2020.1752511 32264724 PMC8143224

[B72] Sharoar M. G. Palko S. Ge Y. Saido T. C. Yan R. (2021). Accumulation of saposin in dystrophic neurites is linked to impaired lysosomal functions in Alzheimer's disease brains. Mol. Neurodegener. 16 (1), 45. 10.1186/s13024-021-00464-1 34215298 PMC8254260

[B73] Shulman L. M. (2007). Gender differences in Parkinson's disease. Gend. Med. 4 (1), 8–18. 10.1016/s1550-8579(07)80003-9 17584622

[B74] Sliter D. A. Martinez J. Hao L. Chen X. Sun N. Fischer T. D. (2018). Parkin and PINK1 mitigate STING-induced inflammation. Nature 561 (7722), 258–262. 10.1038/s41586-018-0448-9 30135585 PMC7362342

[B75] Sorice M. (2022). Crosstalk of autophagy and apoptosis. Cells 11 (9), 1479. 10.3390/cells11091479 35563785 PMC9102887

[B76] Sun W. L. (2016). Ambra1 in autophagy and apoptosis: implications for cell survival and chemotherapy resistance. Oncol. Lett. 12 (1), 367–374. 10.3892/ol.2016.4644 27347152 PMC4906955

[B77] Suresh S. N. Chakravorty A. Giridharan M. Garimella L. Manjithaya R. (2020). Pharmacological tools to modulate autophagy in neurodegenerative diseases. J. Mol. Biol. 432 (8), 2822–2842. 10.1016/j.jmb.2020.02.023 32105729

[B78] Sveinbjornsdottir S. (2016). The clinical symptoms of Parkinson's disease. J. Neurochem. 139 (Suppl. 1), 318–324. 10.1111/jnc.13691 27401947

[B79] Trist B. G. Hare D. J. Double K. L. (2019). Oxidative stress in the aging substantia nigra and the etiology of Parkinson's disease. Aging Cell. 18 (6), e13031. 10.1111/acel.13031 31432604 PMC6826160

[B80] Tu H. Y. Yuan B. S. Hou X. O. Zhang X. J. Pei C. S. Ma Y. T. (2021). α-synuclein suppresses microglial autophagy and promotes neurodegeneration in a mouse model of Parkinson's disease. Aging Cell. 20 (12), e13522. 10.1111/acel.13522 34811872 PMC8672776

[B81] Umeh C. C. Perez A. Augustine E. F. Dhall R. Dewey R. B. Jr. Mari Z. (2014). No sex differences in use of dopaminergic medication in early Parkinson disease in the US and Canada - baseline findings of a multicenter trial. PLoS One 9 (12), e112287. 10.1371/journal.pone.0112287 25486269 PMC4259292

[B82] Urano Y. Mori C. Fuji A. Konno K. Yamamoto T. Yashirogi S. (2018). 6-Hydroxydopamine induces secretion of PARK7/DJ-1 via autophagy-based unconventional secretory pathway. Autophagy 14 (11), 1943–1958. 10.1080/15548627.2018.1493043 30112966 PMC6152502

[B83] Wong Y. C. Krainc D. (2017). α-synuclein toxicity in neurodegeneration: mechanism and therapeutic strategies. Nat. Med. 23 (2), 1–13. 10.1038/nm.4269 PMC8480197 28170377

[B84] Yan W. Zhang T. Kang Y. Zhang G. Ji X. Feng X. (2021). Testosterone ameliorates age-related brain mitochondrial dysfunction. Aging (Albany NY) 13 (12), 16229–16247. 10.18632/aging.203153 34139672 PMC8266321

[B85] Young L. J. Pfaff D. W. (2014). Sex differences in neurological and psychiatric disorders. Front. Neuroendocrinol. 35 (3), 253–254. 10.1016/j.yfrne.2014.05.005 24882637

[B86] Yu L. Liao P. C. (2000). Sexual differences and estrous cycle in methamphetamine-induced dopamine and serotonin depletions in the striatum of mice. J. Neural Transm. (Vienna) 107 (4), 419–427. 10.1007/s007020070084 11215753

[B87] Zhang J. Culp M. L. Craver J. G. Darley-Usmar V. (2018). Mitochondrial function and autophagy: integrating proteotoxic, redox, and metabolic stress in Parkinson's disease. J. Neurochem. 144 (6), 691–709. 10.1111/jnc.14308 29341130 PMC5897151

[B88] Zhang K. Zhu S. Li J. Jiang T. Feng L. Pei J. (2021b). Targeting autophagy using small-molecule compounds to improve potential therapy of Parkinson's disease. Acta Pharm. Sin. B 11 (10), 3015–3034. 10.1016/j.apsb.2021.02.016 34729301 PMC8546670

[B89] Zhang Z. Yang X. Song Y. Q. Tu J. (2021a). Autophagy in Alzheimer's disease pathogenesis: therapeutic potential and future perspectives. Ageing Res. Rev. 72, 101464. 10.1016/j.arr.2021.101464 34551326

[B90] Zhao X. Chen Y. Wang L. Li X. Chen X. Zhang H. (2020). Associations of ATG7 rs1375206 polymorphism and elevated plasma ATG7 levels with late-onset sporadic Parkinson's disease in a cohort of Han Chinese from southern China. Int. J. Neurosci. 130 (12), 1206–1214. 10.1080/00207454.2020.1731507 32065549

